# Determining addition pathways and stable isomers for CF_3_ functionalization of endohedral Gd@C_60_

**DOI:** 10.1098/rsos.180588

**Published:** 2018-09-05

**Authors:** Chris Ewels, Jeremy Rio, Hiroyuki Niwa, Haruka Omachi, Hisanori Shinohara, Mark Rayson, Patrick Briddon

**Affiliations:** 1 Institut des Materiaux Jean Rouxel (IMN), Université de Nantes, CNRS UMR6502, 2 Rue de la Houssiniere, BP32229, Nantes 44322, France; 2 Department of Chemistry, Graduate School of Science, Nagoya University, Chikusa 464-8602, Nagoya, Japan; 3 Institute for Advanced Research, Nagoya City University, Chikusa 464-8602, Nagoya, Japan; 4 School of Engineering, University of Newcastle, Newcastle upon Tyne NE1 7RU, UK

**Keywords:** metallofullerenes, gadolinium, density functional calculations, addition pathways

## Abstract

Using density functional theory approaches, we follow the sequential addition of CF_3_ functional groups to the surface of the metallic endofullerene species Gd@C_60_. The presence of gadolinium in the interior of the cage strongly influences the addition sequence. The calculations are able to successfully identify end points in the addition sequence at Gd@C_60_(CF_3_)_*n*_, *n* = 3 and two isomers at *n* = 5, in predictive agreement with experiment. Inverting the algorithm to determine the most labile groups also identifies the correct positively charged $\text{Gd@}{\text{C}}_{60}{\left(\text{C}{\text{F}}_{3}\right)}_{4}^{+}$ isomer, as confirmed by experimental mass spectra. The importance of surface mobility, notably at later stage addition, is discussed.

## 1. Introduction

Functionalization of carbon fullerene molecules is now a mature field, with chemical group addition to the cage surface used to impart new functionality and resulting in species with application in diverse areas, notably light harvesting [1]. Another motivation for functionalization of fullerene cage surfaces is to stabilize the cages, notably for carbon cages that do not obey the isolated pentagon rule (IPR). In these cases, functionalization removes dangling bond or gap states, opening the energy gap between highest occupied (HOMOs) and lowest unoccupied (LUMOs) molecular orbitals and stabilizing the species [2–5]. In a similar way, functionalization has been proposed as a route to stabilization of certain metallic endofullerenes [5,6]. Introduction of a metallic atom within the cage can sometimes result in a locally reactive cage surface, which results in aggregation and hinders isolation and separation of the metallofullerene species. This is particularly important when there is covalent orbital interaction between the metallic species and the *p_z_*-orbitals of the surrounding cage.

An important recent case in point is the encapsulation of a gadolinium atom within the *I_h_*-C_60_ fullerene cage. This species has only recently been successfully isolated and its crystal structure identified [1] through a process of *in situ* trifluoromethylation during arc-discharge synthesis of the metallofullerenes [7]. The resulting CF_3_ functionalized species have wide HOMO–LUMO gaps not much reduced from the parent C_60_, enabling their crystallization and characterization. In that study, we briefly reported supporting density functional theory (DFT) calculations exploring isomer formation. Insertion and stabilization of heavy atoms like gadolinium inside the C_60_ molecule is of interest for a variety of applications, for example in superconductivity [8] or biomedical applications, in which its very great magnetic moment makes it a promising candidate as a contrast agent for magnetic resonance imaging [9].

In the current publication, we explore the functionalization process via such DFT calculations in significantly more depth, and discuss the importance and limitations of the results and methods employed, as well as extending them to removal of labile surface species through positive ionization. The idea here is to use DFT modelling techniques to determine the position of the functional groups used to stabilize the structure *without experimental input*. A full exploration of all possible isomers of Gd@C_60_(CF_3_)*_n_*, *n* = 1 … 6 (or more), would be impossible with currently available computing resources. Simply assuming that different combinations of surface carbon sites are functionalized by CF_3_ groups results in an enormous number of potential isomers. Additional variability is present in this case owing to the potential rotational variance of the CF_3_ group, as well as the spatial location of the Gd in the cage interior. Clearly, such a problem is not tractable with a simple ‘brute force’ approach to calculating all possible variants.

We developed a potential solution to this problem several years ago by making the assumption that, in most cases, addition occurs *sequentially*, and is not followed, at least at low surface coverages, by surface rearrangement [2,3]. The current publication is the first time that the approach has been attempted as a predictive tool with no prior experimental data available. Assuming sequential addition, we first model all possible structures with the addition of a single CF_3_ group, selecting the most stable from these 60 calculations (symmetry can be used to reduce this number slightly, but the presence of Gd limits this compared with pristine *I_h_*-C_60_). The most energetically favourable structure for Gd@C_60_(CF_3_) is then used to add a second CF_3_ at all possible addition sites, generating a further 59 possible structures, all of which were optimized. Again the most stable structure was taken for Gd@C_60_(CF_3_)_2_ and the process continued for further CF_3_ addition. This approach is detailed further in [10], and has previously been successfully applied to determine fluorinated isomers of C_60_ [11] and chlorinated isomers of C_70_ [12] in good agreement with experiment. In cases where two isomers were closer than 2.3 kcal mol^−1^ (0.1 eV) in energy and not related by symmetry, both were used as starting points for subsequent CF_3_ addition. In this way, it is possible to trace a full additional sequence ‘tree’. In total, around 1000 isomers were geometrically optimized for the study. Furthermore, we present supporting experimental mass spectrometry data on charged Gd@C_60_(CF_3_)*_n_* species.

### 1.1. C_60_(CF_3_)_*n*_

Trifluoromethylation has been previously used as a route to functionalize pristine C_60_, representing some of the most studied fullerene derivatives [13–19]. There are around 30 fullerene-(CF_3_)*_n_* species with measured X-ray structures and many others with different physicochemical characterizations, of which nearly 30 C_60_(CF_3_)*_n_* derivatives have been described ([12] and references therein, and the electronic supplementary material of [20]). All of these involve the addition of an even number of trifluoromethyl groups, from *n* = 2 to *n* = 18, with multiple isomers identified, notably for *n* = 8 and *n* = 10. Functionalization of C_60_ is typically performed post-growth, for example through exposure of C_60_ to gaseous CF_3_I [12] or silver(I) trifluoroacetate [17] at elevated temperatures (greater than 300°C, the decomposition temperature of CF_3_I), although there are also studies showing the formation of species up to *n* = 8 from arc-electric fullerene growth using a polytetrafluoroethylene (PTFE)-doped graphite powder electrode [21].

The multiple *n* = 2–18 isomers show different addition pattern behaviours [12]. For *n* = 2, CF_3_ adds in the para- position across a hexagon [13–17]. The *n* = 6 isomers include 1,6,9,12,15,18-C_60_(CF_3_)_6_, a structure in which a pentagon is isolated from the rest of the cage with CF_3_ groups on all back-bond sites, and a sixth CF_3_ group on one of the pentagon sites [18]. Structure 1,6,11,18,24,27-C_60_(CF_3_)_6_ bears some similarity to the CF_3_-functionalized endofullerene structures discussed below, namely CF_3_ groups occupy four of the five back-bond sites around a pentagon [16,17]. In the case of 1,6,11,18,24,27-C_60_(CF_3_)_6_, the fifth and sixth CF_3_ functionalization sites lie close nonetheless, the fifth CF_3_ group sitting on a carbon neighbouring the unfunctionalized back-bond site and the sixth CF_3_ group sitting on the para- site of the hexagon shared with the fifth group. This motif, of four CF_3_ groups sitting on back-bond sites around a shared pentagon, is in general repeated in most of the higher-order isomers (*n* = 8–18), and results in two double bonds sitting on the central pentagon [12].

A more recent theoretical study has analysed these in terms of thermodynamics and kinetics using a genetic protocol to construct regio-isomers [20]. The authors show that two functionalization processes appear to compete: direct addition/abstraction of CF_3_ groups, for which a purely thermodynamic model is sufficient to identify most products, and slower surface addend migration processes for which non-equilibrium kinetic modelling of addition sequences is required.

As we will see below, the introduction of a metal atom within the fullerene sphere significantly modifies this behaviour. There are two computational studies of trifluoromethylation of metallofullerenes in the literature to our knowledge, the first concerning Y@C_60_(CF_3_)*_n_* [22], the second La@C_60_(CF_3_)*_n_* [23]. The first study, combined with experimental mass spectra studies, modelled both Y@C_60_(CF_3_) and Y@C_60_(CF_3_)_3_, the latter structure having three CF_3_ groups on back bonds around the same shared pentagon (equivalent to the top-right Schlegel diagram in figure 6), for which they found low to moderate HOMO–LUMO gaps of 0.30 eV and 0.81 eV, respectively. The second study explored La@C_60_(CF_3_)*_n_* for *n* = 0–5, with 6–10 regio-isomers selected by hand for each *n*. At *n* = 3 the authors identified an equivalent CF_3_ arrangement to Y@C_60_(CF_3_)*_n_* above as the thermodynamically preferred configuration, along with a high-symmetry C_2v_ configuration for *n* = 4. The structures for *n* = 3 and *n* = 4 are not identified as stable addition end points in our calculations, for reasons discussed below.

## 2. Method

DFT calculations were performed under the local spin density approximation, implemented in the AIMPRO code [24–27]. Relativistic pseudopotentials were included via the Hartwigsen–Goedecker–Hütter scheme [28]. The basis consisted of Gaussian function sets multiplied by polynomial functions including all angular momenta up to maxima *p* (*l* = 0, 1), *d* (l = 0, 1, 2) and *f* (*l =* 0–3) [29]. For carbon a pdddp basis set was used, resulting in 38 independent functions, for Gd an fffff basis (90 independent basis functions) and for fluorine ddpp (28 independent functions). Calculations were fully spin polarized with spin relaxation. Periodic boundary conditions at the gamma point were applied, with cell size large enough to avoid interaction between neighbouring fullerenes. A system-dependent plane wave energy cut-off of 300 Ha (Ha, Hartree energy) and a non-zero electron temperature of kT = 0.04 eV for electronic-level occupation were taken. Atomic positions were geometrically optimized until the maximum atomic position change in a given iteration dropped below 10^–6^ a_0_ (a_0_, Bohr radius). Diffusion barrier calculations were obtained via the climbing nudged elastic band between fully optimized end points. Charge and spin states of individual atoms were obtained using Mulliken population analysis. Symmetry was deliberately broken, and none imposed during optimization, to ensure the structures were not symmetrically over-constrained. In total around 1000 structures were geometrically optimized for this study. For comparison we also optimized key stable structures at the unrestricted GGA level of theory (using PBE [30] and Krack pseudopotentials [31]), obtaining almost identical structures and HOMO–LUMO gaps with both functionals.

The enthalpies of reaction are calculated using (CF_3_)_2_ as the reference state for the trifluoromethyl groups:

$$\text{Gd@}{\text{C}}_{60}{\left(\text{C}{\text{F}}_{3}\right)}_{n-1}+1\;/\;2{\left(\text{C}{\text{F}}_{3}\right)}_{2}\to \text{Gd@}{\text{C}}_{60}{\left(\text{C}{\text{F}}_{3}\right)}_{n}.$$

We take the enthalpy difference as an indication of the relative stability in large part due to computational expediency. This is something of an assumption, although, given the similarity in structural motifs, we might expect the difference in entropy between isomers to be small. We note also that the HOMO–LUMO gap in our stable structures is in general much larger than that in other isomers (as discussed below), even though this is not used as a selection criterion. Kinetic stability is also discussed further below.

## 3. Results

### 3.1. Gd@C_60_

**Figure 1.**
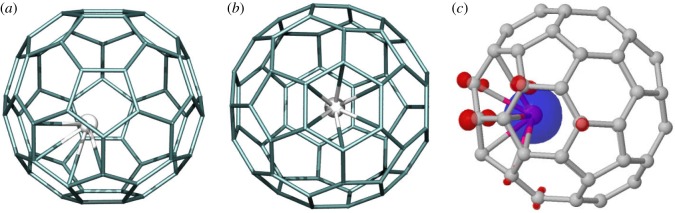
(*a*,*b*) Gd@C_60_, showing the close proximity of Gd to the carbon cage (six Gd–C bonds of average length 2.407 Å). System net magnetic moment of 6.72 µ_B_. (*c*) Spin distribution showing excess spin-up (spin-down) in blue (red); cut-off 0.007e bohr^−3^.

In Gd@C_60_, the Gd atom drifts away from the fullerene centre, sitting instead below a hexagon with a Gd–C average bond length of 2.407 Å (figure 1). There is a net calculated spin on Gd of 7.14 µB and total system spin of 6.71 µB. This is consistent with previous theoretical calculations [32], which also found that Gd is stable when adjacent to a hexagon centre with Gd–C distances of 2.38–2.41 Å and spin density of 6.74 µ_B_ on Gd. We also tested Gd inside the C_2v_–C_60_ isomer no. 1809 (structurally obtained from *I_h_*-C_60_ through a 90° bond rotation of a single C–C shared hexagon bond about the bond centre, resulting in two paired pentagons on the fullerene surface). While this isomer is 36.9 kcal mol^−1^ less stable than *I_h_*-C_60_ when empty, this energy difference drops to only 20.45 kcal mol^−1^ in the presence of encapsulated Gd. Hence, while Gd appears able to stabilize non-IPR isomers, the energy difference is still sufficient such that we do not expect a significant population of Gd@#1809-C_60_ metallic endofullerene in the sample. This is consistent with the experimental X-ray data, which show only Gd@*I_h_*-C_60_ in the trifluoromethylated species [4].

### 3.2. Gd@C_60_(CF_3_)*_n_*, *n* = 1–5

The CF_3_ addition sequence in the following section is predicated on a number of assumptions. Firstly, we assume the CF_3_ groups add sequentially, and, once the stable addition site has been found, they are not subsequently surface mobile. Secondly, we compare the CF_3_ binding energy with that of a (CF_3_)_2_ molecular species, on the assumption that, once this comparative binding energy becomes positive, there will be a net energetic driving force for CF_3_ species to combine and leave the fullerene surface, and hence addition will cease. Finally, we assume that, unless the two most stable symmetry-distinct isomers have calculated energies within 0.1 eV of each other (approx. 15–20% of the CF_3_ binding energy), the second most stable isomer will be statistically so unlikely to form that it can be ignored. When two isomers are close in energy, we take both as potential starting points for subsequent CF_3_ addition, allowing us to trace multiple addition pathways. We will show below that these assumptions lead to a remarkable predictive agreement with experimentally determined isomers.

**Figure 2.**
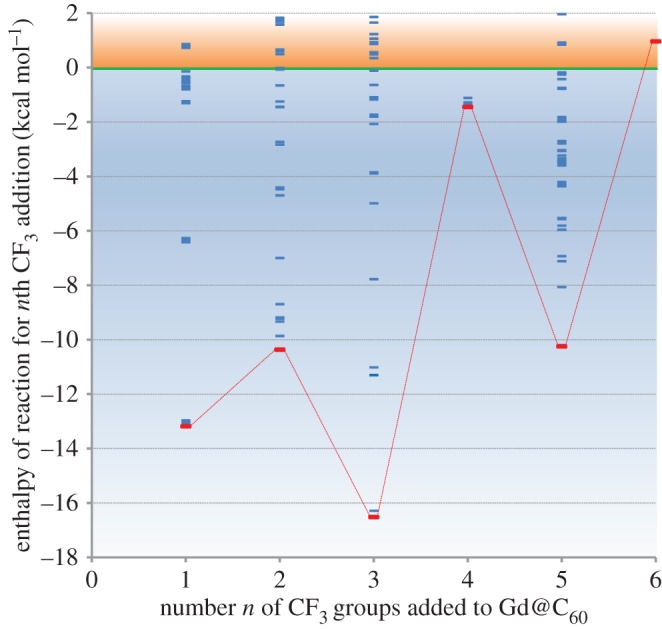
Enthalpy of reaction for CF_3_ addition to Gd@C_60_ in kcal mol^−1^, following the reaction Gd@C_60_(CF_3_)*_n_*_−1_ + 1/2 (CF_3_)_2_ → Gd@C_60_(CF_3_)*_n_*. Each blue line represents an optimized isomer structure; most stable isomers are marked in red and connected via dotted red lines. Positive enthalpies (orange zone on plot) indicate that the CF_3_ addition is not stable.

Figure 2 shows a plot of the binding energies for CF_3_ in the most stable addition sequence to Gd@C_60_. Note that the axis does not show the average binding energy for each CF_3_ group at this surface density, but rather the energy change involved in the addition of a new CF_3_ group.

The plot shows that CF_3_ addition is stable up to (CF_3_)_5_; above this addition becomes endothermic. Odd-numbered addition is energetically the most favourable, and notably addition to form Gd@C_60_(CF_3_)_3_ is extremely favourable, with (CF_3_)_4_ acting as a barrier before the addition sequence completes at (CF_3_)_5_. Thus these calculations suggest that *n* = 3 and *n* = 5 are likely to be stable ‘magic number’ species.

The system spin fluctuates between 7.0 µ_B_ for all odd-numbered additions (including the stable *n* = 3 and *n* = 5) and 6.4–7.3 µ_B_ for even-numbered additions. We note that figure 2 only shows a zoom around the lowest energy isomers, with significantly less stable structures found with enthalpies of reaction of up to +32 kcal mol^−1^.

**Figure 3.**
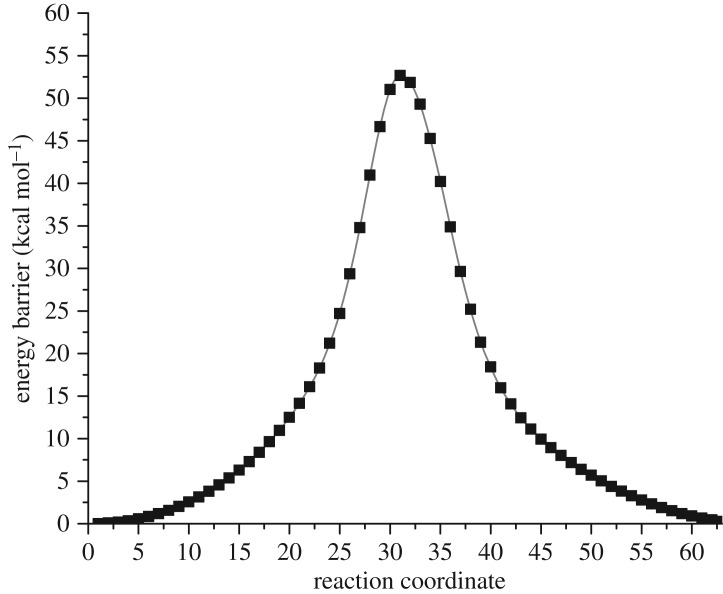
Calculated reaction barrier for migration of CF_3_ between neighbouring carbon sites on the surface of Gd@C_60_ (kcal mol^−1^).

For the initial *n* = 1 addition, there are around 20 structures with energies within 2 kcal mol^−1^ of each other. This is because Gd is not constrained during optimization and alters its position within the cage, rendering all structures roughly equivalent when the Gd atom sits beneath a hexagon with a shift towards a neighbouring pentagon, and addition occurs on one of the back bonds of this pentagon. To test our initial assumption that CF_3_ groups will not be surface mobile, we calculated the diffusion barrier for CF_3_ to move to a neighbouring site, using a nudged elastic band method. The results (figure 3) show that the barrier is surprisingly high at 52.7 kcal mol^−1^, with the CF_3_ essentially de-bonding and re-bonding to the neighbouring site, confirming that CF_3_ will not be surface mobile.

**Figure 4.**
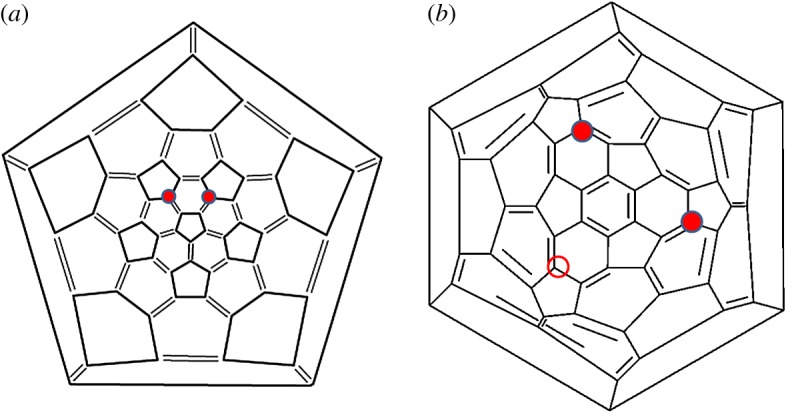
Schlegel projection diagrams showing the two most stable addition sites for *n* = 2, Gd@C_60_(CF_3_)_2_, with CF_3_ groups added at sites marked with red circles: (*a*) most stable addition site; (*b*) structure 1.0 kcal mol^−1^ less stable. Empty circle shows a third equivalent addition site by symmetry. In each case, the Gd atom sits beneath the central hexagon in (*b*).

For the *n* = 2 second addition, two distinct sites can be identified. The most stable addition is at one of the two symmetrically equivalent sites, continuing CF_3_ addition around the back bonds of the pentagon neighbouring the Gd (figure 4*a*). However, a second addition site, shown in figure 4*b*, is only 1.0 kcal mol^−1^ less stable, where Gd migrates once more towards the central hexagon. The plot in figure 2 shows continued addition to the structure shown in figure 4*a*.

**Figure 5.**
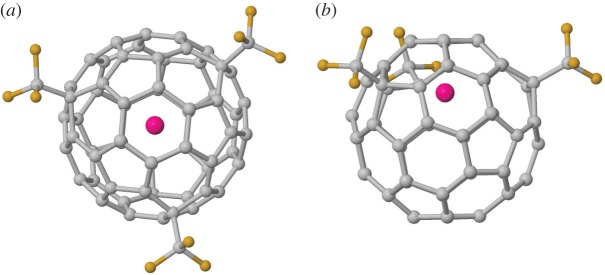
Predicted stable Gd@C_60_(CF_3_)_3_ isomer with C_3_ symmetry. (*a*) Side and (*b*) top view.

**Figure 6.**
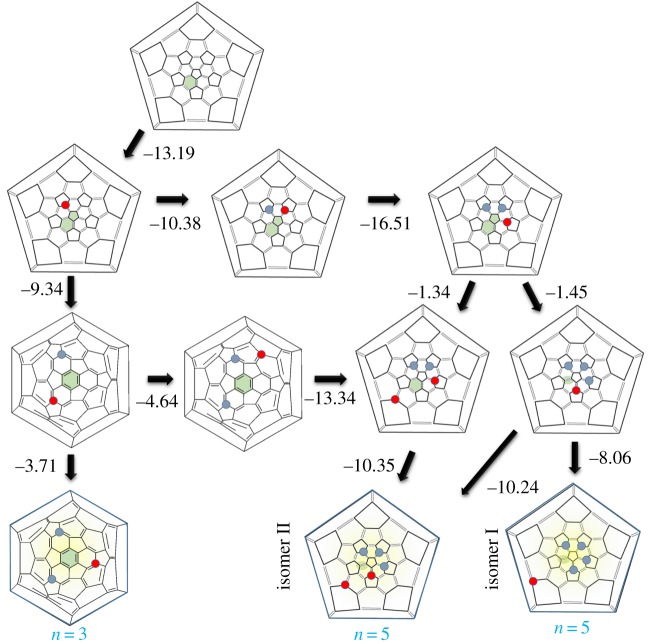
Schlegel projections showing calculated CF_3_ addition pathways to Gd@C_60_ (top) to stable trifluoromethylated species (bottom). Previous addition sites are marked with blue circles; new addition sites are marked with red circles. Green shading indicates the location of the Gd atom. Numbers indicate energy release in kcal mol^−1^ (from figure 2). All pathways are shown where isomers are within 2.3 kcal mol^−1^ of the most stable. Fullerenes at the bottom of the figure where further addition is unfavourable have shaded backgrounds and a dark outline, and represent stable end points of a reaction pathway (adapted from Nakagawa *et al*. [6]).

Before continuing with the primary addition sequence, we first explore further CF_3_ addition to the *n* = 2 isomer shown in figure 4*b*. Once again there are two addition sites quite close in energy for *n* = 3. The most stable site is shown as an empty circle in figure 4*b* and results in a C_3_ symmetry isomer Gd@C_60_(CF_3_)_3_ as shown in figure 5. Further addition to this structure is endothermic, showing that it is a stable end structure for addition and a predicted magic number. The second possible addition site to figure 4*b*, 0.93 kcal mol^−1^ less stable than that shown in figure 5, has only one stable subsequent CF_3_ addition site for *n* = 4, which returns it to the primary addition sequence (shown in figure 6 below). Thus in conclusion, this side branch at *n* = 2 offers two alternatives, one of which results in a new predicted stable C_3_ symmetry isomer of Gd@C_60_(CF_3_)_3_, while the other continues to add CF_3_ and returns to the primary addition pathway.

**Figure 7.**
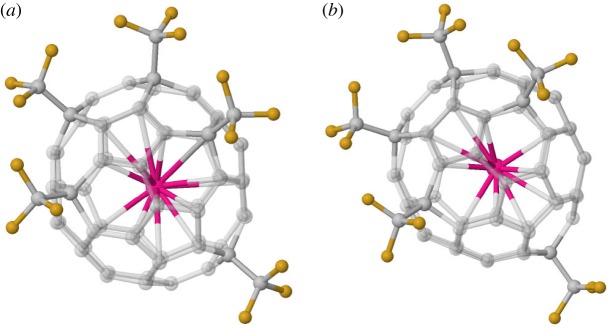
Calculated stable isomer structures for Gd@C_60_(CF_3_)_5_: (*a*) $\text{Gd@}{\text{C}}_{60}{\left(\text{C}{\text{F}}_{3}\right)}_{5}^{\left(I\right)}$ and (*b*) $\text{Gd@}{\text{C}}_{60}{\left(\text{C}{\text{F}}_{3}\right)}_{5}^{\left(\mathrm{II}\right)}$. Gd, pink; C, grey; F, yellow.

The entire addition pathway, including the bifurcating path at *n* = 2, is shown in figure 6. The primary addition pathway, shown around the right edge of the diagram, continues with CF_3_ group addition to the back bonds of a single pentagon. The exception to this occurs for the final addition step at *n* = 5, where, instead of completing the pentagon enclosure, the final CF_3_ group adds at a more distant site corresponding to the secondary site favoured at *n* = 2. An alternative *n* = 5 isomer is also close in energy, where the CF_3_ group sits slightly further away. Together these two structures represent the predicted stable ‘magic number’ structures for Gd@C_60_(CF_3_)_5_ and are shown in figure 7. We note that it is extremely endothermic to attempt to complete the sequence of five CF_3_ groups around the pentagon; of all possible addition sites for *n* = 5 on the cage, this is the second *least* stable site and is 20.54 kcal mol^−1^ less stable than isomer I shown in figure 6.

The calculations are in excellent agreement with experimental observation. The three primary observed species after CF_3_ functionalization of Gd@C_60_ are Gd@C_60_(CF_3_)_3_, and the two isomers of Gd@C_60_(CF_3_)_5_ identified in figure 7 [4]. X-ray crystallography confirms that the experimental isomers indeed correspond to those identified here, including the underlying location of Gd. Additionally, the most stable isomer (figure 7*a*) is found in roughly double the quantities of the isomer shown in figure 7*b*, consistent with the calculations, which both show it to be the energetically most stable isomer, and also the isomer representing the end point of the majority of the addition sequences. We note that the calculated CF_3_ migration barrier between the $\text{Gd@}{\text{C}}_{60}{\left(\text{C}{\text{F}}_{3}\right)}_{5}^{\left(I\right)}$ and $\text{Gd@}{\text{C}}_{60}{\left(\text{C}{\text{F}}_{3}\right)}_{5}^{\left(\mathrm{II}\right)}$ isomer is only 24.7 kcal mol^−1^, and hence it is feasible at room temperature that these two isomers may interchange over a time scale of seconds.

There is a clear pattern in the addition behaviour. The bifurcation in addition routes at *n* = 2 is linked to the position of the Gd within the cage. The separated addition route (lower line, figure 6) corresponds to Gd remaining located next to its initial hexagonal site. The primary addition route (top line, figure 6) corresponds to Gd migrating closer to the central pentagon and its associated functional groups, in most cases located under the pentagon–hexagon bond, and at the end of the addition sequence, under the neighbouring hexagon–hexagon bond. Thus Gd appears to play a dual role when selecting CF_3_ addition sites, both acting as charge donor and enhancing surface charge localization, but also through the strong hybridization between the Gd valence states and the C 2p states of the fullerene. This suggests that we would expect different addition sequences for non-covalently bound endohedral +3 oxidation species.

It is important to note that the structures predicted by this method do not necessarily correspond to the thermodynamically most stable regio-isomers. For example for *n* = 3, the structure top right of figure 6 is 12.81 kcal mol^−1^ more stable than the structure at the bottom left (in agreement with previous calculations for La@C_60_(CF_3_)_3_ [23]), yet it is the bottom-left structure that we predict to be experimentally observable (as confirmed by experiment [6]). In this case, the thermodynamically more stable regio-isomer is not stable because it is simply an intermediate structure en route to further CF_3_ functionalization. This shows that kinetic effects during functionalization cannot be ignored, as has also been shown for some pristine C_60_ trimethylfluorinated species [20].

### 3.3. Role of Gd in the addition sequence: metallocene behaviour

Inclusion of Gd within the C_60_ cage significantly alters the functionalization behaviour of the fullerene. Pristine C_60_ shows a wide range of isomers upon trifluoromethylation, with comparatively high surface densities (up to *n* = 18 CF_3_ functional groups and higher; see Introduction). By contrast, Gd@C_60_ shows only the three stable isomers reported here for *n* = 3, 5(I) and 5(II). As the functionalization methods are different, we cannot fully exclude the possibility of higher surface coverage species if, for example, Gd@C_60_(CF_3_)_5_ is subsequently exposed to CF_3_I at 550°C; however, the calculations suggest this should remain unlikely as such processes would be endothermic compared with (CF_3_)_2_ production.

As might be expected, while pristine C_60_ is only stable with even-numbered *n*, Gd@C_60_ prefers odd-numbered *n* due to its +3 charge state and hence initial open-shell configuration of the unfunctionalized metallofullerene [12].

Some insight into the behaviour and bonding of the Gd can be taken from Gd metallocene complexes. Gd is known to form stable metallocene species, notably the Tris(cyclopentadienyl)gadolinium(III) complex Gd(C_5_H_5_)_3_ consisting of three pentagonal cyclopentadienyl units surrounding the central Gd atom. Other species include the bromine-stabilized metallocene dimer (Gd(C_5_H_5_)_2_)_2_-Br_2_ [33]. Unlike sandwich metallocenes such as ferrocene, this species has the cyclopentadienyl tilted away from the dimer complex centre, opening up access to the Gd atom to allow complexation with bromine. In both cases, Gd sits surrounded by anionic pentagonal C_5_H_5_ groups.

In our case, by sp^3^ hybridizing the back bonds of the pentagon in Gd@C_60_(CF_3_)_5_, CF_3_ functionalization effectively isolates a structural equivalent of a C_5_H_5_ unit on the fullerene surface. However, a *pair* or *triplet* of these, as energetically preferred by the Gd, is not possible within the confines of the C_60_ cage structure. The closest it can come to this is by positioning the Gd between this semi-isolated pentagon and its nearest pentagon neighbour, beneath the interconnecting bond. The fullerene curvature allows Gd to interact with both canted pentagons. While the first pentagon is surrounded by CF_3_ groups and hence has single back bonds and can reproduce C_5_H_5_ anionic behaviour, the second pentagon is not able to form an equivalent isolated anion because CF_3_ addition becomes energetically unfavourable after *n* = 5. Nonetheless, the remaining single CF_3_ addition sits at sites which begin this process.

Gd sits beneath the interconnecting bond between these two pentagons. The two covalent bonds it would normally form with a halide in (Gd(C_5_H_5_)_2_)_2_-Br_2_ are partially replaced by formation of covalent bonds with this core carbon atom pair (replacing what would be a double bond in a conventional fullerene). Thus this model would suggest that CF_3_ addition allows Gd to recreate an approximation towards a local metallocene-like environment. We note that this double bond replacement is similar to the structure seen in C_60_(CF_3_)_4_O, where, in this case, an epoxide bond is formed on the fullerene exterior of this core carbon atom pair [18].

### 3.4. Positive +1 charge state: $\text{Gd@}{\text{C}}_{60}{\left(\text{C}{\text{F}}_{3}\right)}_{4}^{+}$

It is interesting to explore the energy landscape for the +1 charged system. During laser desorption positive-ion mass spectrum measurements, laser excitation excites and removes one electron. We approached this problem in two distinct ways but arrived at similar conclusions. The first was simply to repeat the addition pathway determination as discussed above for the neutral species, but with the cage in a +1 charge state at each step. When we do so, we obtain a similar addition pathway and stable species, with the exception that the second route towards ${\text{C}}_{3}-\text{Gd@}{\text{C}}_{60}{\left(\text{C}{\text{F}}_{3}\right)}_{3}^{+}$ becomes destabilized. Importantly, addition at *n* = 5 becomes endothermic. Thus the addition pathway is expected to finish at *n* = 4 when in the +1 charge state with Gd@C_60_(CF_3_)_4_^+^, the stable positively charged isomer. As the second addition route is destabilized, the calculations predict only one stable isomer, where all four CF_3_ groups sit at back-bond sites to the same pentagon.

**Figure 8.**
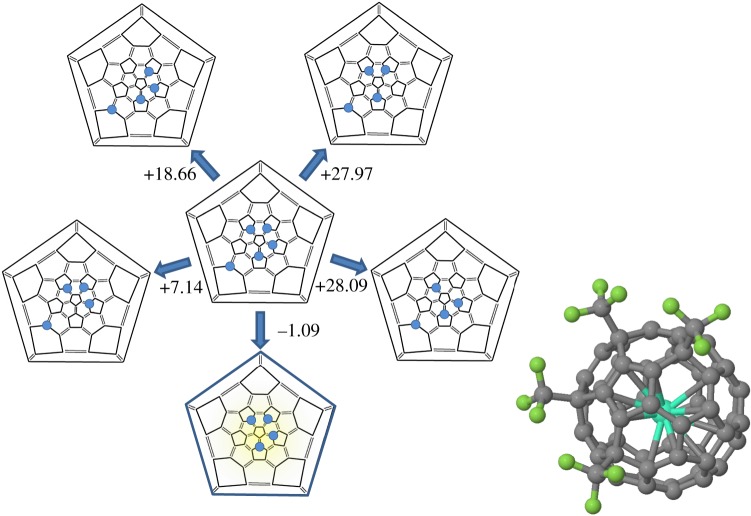
Schlegel diagrams showing the calculated energy for $\text{Gd@}{\text{C}}_{60}{\left(\text{C}{\text{F}}_{3}\right)}_{5}^{+}\to \text{Gd@}{\text{C}}_{60}{\left(\text{C}{\text{F}}_{3}\right)}_{4}^{+}+1\;/\;2{(C{\text{H}}_{3})}_{2}$, removing different CH_3_ groups (kcal mol^−1^); bottom shaded diagram indicates the stable isomer (right), the stable $\text{Gd@}{\text{C}}_{60}{\left(\text{C}{\text{F}}_{3}\right)}_{4}^{+}$ isomer obtained in this way.

A second approach to the problem is to assume that stable neutral Gd@C_60_(CF_3_)_5_ species will be photoexcited and lose an electron. Given that the above calculations show *n* = 5 is unstable in the +1 charge state, we removed each of the CF_3_ functional groups from $\text{Gd@}{\text{C}}_{60}{\left(\text{C}{\text{F}}_{3}\right)}_{5}^{\left(I\right)}$ in the +1 charge state to determine which was most labile (figure 8). Consistent with the sequential addition calculations, removal of the CF_3_ groups around the pentagon is endothermic, and only removal of the more isolated CF_3_ group is exothermic. Thus both sets of calculations suggest there will be one clearly stable species and isomer, $\text{Gd@}{\text{C}}_{60}{\left(\text{C}{\text{F}}_{3}\right)}_{4}^{+}$, in the +1 charge state.

This result can be compared directly to experiment. The full sample preparation method is given in [6]. In brief, a DC arc-discharge chamber with flowing He atmosphere is used to produce raw soot, using graphite rods as electrodes, with Gd impregnation in the anode and PTFE rods held nearby. The resultant soot then undergoes *o*-xylene extraction to exclude amorphous carbon. A TiCl_4_ Lewis acid method [34,35] is then used to exclude empty fullerenes, giving a mixture of metallofullerenes. From these, high-performance liquid chromatography (HPLC) purification using a Buckyprep-M column results in specific Gd@C_60_ derivatives, which can be fractionated out using another Buckyprep column. The data presented here come from the highest intensity column fragment.

**Figure 9.**
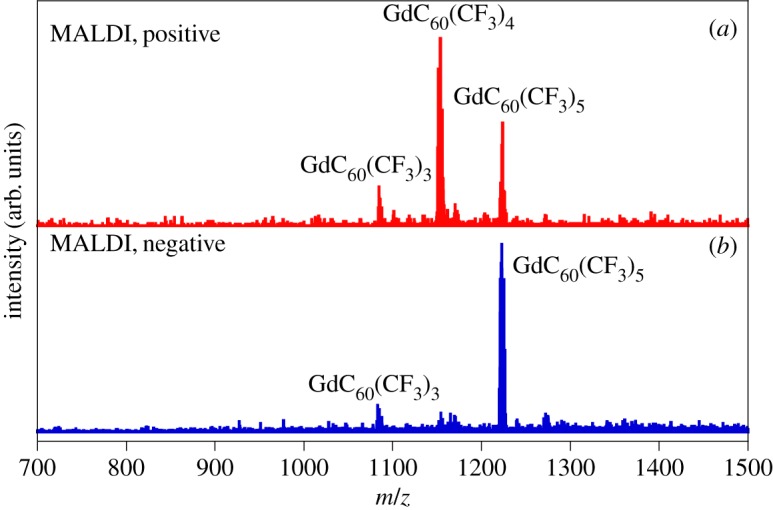
MALDI-TOF data for HPLC second stage separated metallofullerene derivatives, separation using a Buckyprep-M column with toluene eluent at a 21 ml min^−1^ flow rate. Spectrum for the fraction with highest absorbance in the column. (*a*) Positive-ion mode; (*b*) negative-ion mode.

Figure 9 shows matrix-assisted laser desorption/ionization time-of-flight (MALDI-TOF) mass spectra in both positive- and negative-ion modes for separated derivatives of Gd metallofullerenes. Dithranol was used as a MALDI matrix. It should be noted that CF_3_- groups are detached from Gd metallofullerenes at the time of laser desorption and ionization, so that only the intact Gd@C_n_ metallofullerenes are observed. The negative spectrum clearly shows the preferential formation of Gd@C_60_(CF_3_)*_n_*, *n* = 3 and *n* = 5 species as discussed above and in [6]. In contrast in the positive-ion mode, corresponding to the calculations we have performed here, it can be seen that one of the CF_3_ groups in $\text{Gd@}{\text{C}}_{60}{\left(\text{C}{\text{F}}_{3}\right)}_{5}^{+}$ is clearly highly labile, because the concentration of *n* = 5 species is significantly diminished, replaced instead with a high concentration of *n* = 4. Equivalent CF_3_ loss for the *n* = 3 species is not seen, and the *n* = 4 is only present in trace quantities in the negative-ion case. This result is entirely consistent with the calculations presented above.

## 4. Summary and conclusion

In this article, we show that it is possible to successfully predict stable magic number isomers for the trifluoromethylation of Gd@C_60_, by considering the CF_3_ addition process as a sequential addition process to most stable bonding sites. Stable magic number species are identified when subsequent addition is endothermic when compared with (CF_3_)_2_ formation. This approach successfully identifies two Gd@C_60_(CF_3_)_5_ and one Gd@C_60_(CF_3_)_3_ stable isomers which have been identified and characterized experimentally. Equally the method works well for charged species such as the $\text{Gd@}{\text{C}}_{60}{\left(\text{C}{\text{F}}_{3}\right)}_{4}^{+}$ cation. The CF_3_ bonding arrangement and Gd positioning can be rationalized within the framework of a Gd metallocene picture.

The trifluoromethyl functionalization behaviour of Gd@C_60_ is very different from that of pristine C_60_. Along with a switch from addition of even to odd numbers of groups, the wide range of isomers for pristine C_60_(CF_3_)*_n_* is reduced to only three for Gd@C_60_(CF_3_)*_n_*, and maximum coverage is significantly reduced to only five CF_3_ groups. Different addition patterns are observed, although there is some motif similarity with functionalization of back-bond sites around a shared pentagon.

An interesting future study would be to repeat the CF_3_ addition calculations for a sequence of related systems, namely (i) a neutral C_60_, (ii) C_60_ with a +3 charge state but no metal atom, and (iii) C_60_ with a +3 non-covalently bound endohedral metal atom such as Al. Such a sequence of calculations would allow separation of the effects associated with charge transfer, charge location and covalent binding with Gd on the resultant addition sequence. The approach should also be easily adaptable to related systems such as Gd@C_74_(CF_3_)*_n_*, discussed elsewhere in this issue [36].

## Supplementary Material

Ewels_Structures_ESM.tar.gz.png

## Data accessibility

All structures (in *xyz* format) discussed in this article are provided in a .tar file in the electronic supplementary material associated with this article, along with ASCII files tabulating the calculated total energy of each structure in Hartrees.
