# Supplementary figures and images for: Determining addition pathways and stable isomers for CF3 functionalization of endohedral Gd@C60
Source: R Soc Open Sci. 2018 Sep 5;5(9):180588. doi: 10.1098/rsos.180588 (PMC6170591; doi:10.1098/rsos.180588)

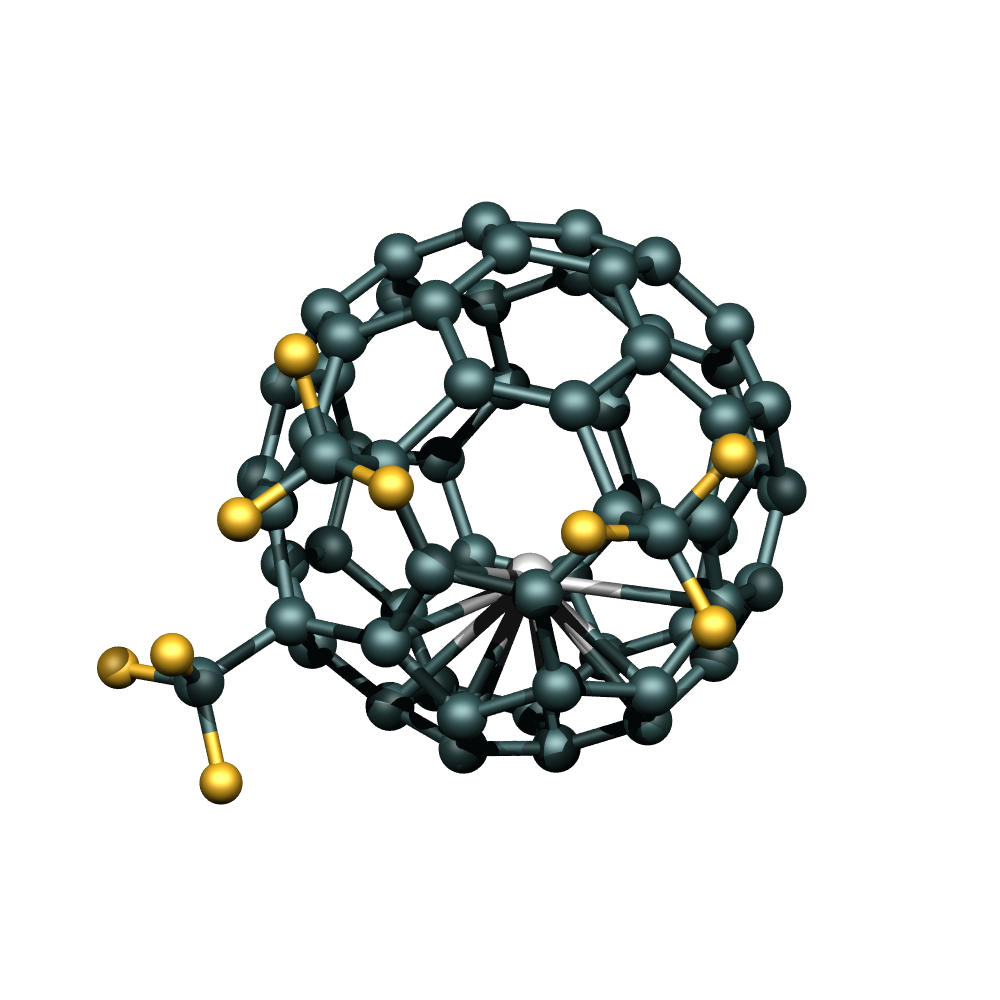

Supplement: Ewels_Structures_ESM.tar.gz.png [file rsos180588supp1.gz › CF3_open_data/neutral/3/21/21.jpg]
